# Effects of passive smoking and its duration on the prevalence of prediabetes and type 2 diabetes mellitus in Chinese women

**DOI:** 10.18632/aging.103217

**Published:** 2020-05-26

**Authors:** Juan Wu, Gui Pan, Yan-Ting Huang, Deng-Ke Liu, Hai-Xia Zeng, Xiao-Jun Zhou, Xiao-Yang Lai, Jian-Ping Liu

**Affiliations:** 1 Department of Endocrinology, The Second Affiliated Hospital of Nanchang University, Nanchang 330000, Jiangxi, China; 2 Department of Pulmonary and Critical Care Medicine, The Second People’s Hospital of Jingdezhen, Jingdezhen 100191, Jiangxi, China; 3 School of Public Health, Nanchang University, Nanchang 330000, Jiangxi, China

**Keywords:** passive smoking, prediabetes, impaired glucose tolerance, type 2 diabetes mellitus, prevalence

## Abstract

Several studies have shown that active smoking is a risk factor for type 2 diabetes mellitus (T2DM). However, the effects of passive smoking on T2DM remains unknown. In this study, we investigated the effects of passive smoking and its duration on the prevalence of prediabetes and T2DM. According to passive smoking status, participants were divided into Group A (passive smokers) and Group B (controls). Furthermore, Group A was divided into three subgroups according to the duration of passive smoking: Group A1 (≤10 years), Group A2 (10–20 years), and Group A3 (>20 years). We found that the prevalence of impaired glucose tolerance (IGT) in Group A (26.6%), Group A2 (28%), and Group A3 (37.8%) was significantly higher than that in Group B (19.6%), and the prevalence gradually increased with an increase in the duration of passive smoking. Multiple logistic regression analysis showed that passive smoking for >10 years was a risk factor for impaired fasting glucose (IFG), IGT, and T2DM. Therefore, passive smoking not only increases the prevalence of IGT in a time-dependent manner, but also a risk factor for IFG, IGT, and T2DM when its duration is over 10 years.

## INTRODUCTION

The global diabetes atlas released by the International Diabetes Federation shows that the number of adult diabetes patients in 2019 was 463 million, excluding 232 million undiagnosed cases, one fifth of them are over 65 years old. In addition, the number of prediabetes patients was approximately 374 million. According to the current growth trend, approximately 700 million people will have diabetes by 2045 [1]. A pooled analysis based on 751 studies revealed that the age-standardized global prevalence of adult diabetes from 1980 to 2014 increased from 4.3% to 9.0% in men and 5.0% to 7.9% in women [2]. In the same period, the prevalence rates of T2DM and prediabetes in China were 10.4% and 35.7%, respectively [3]. Therefore, there is an urgent need for identifying risk factors that contribute to the development of prediabetes and T2DM. In particular, definite and reversible factors must be identified to help reduce the development of T2DM and its complications worldwide.

Smoking is a known risk factor for several chronic diseases (such as chronic obstructive pulmonary disease, lung cancer, ischemic heart disease, and stroke) [4–6]. Although smoking can be avoided, it contributes to the global economic burden of diseases. Moreover, the daily smoking rate has significantly reduced worldwide; however, the crude number of smokers is still increasing with an increase in the population [7]. As a result, the number of passive smokers is also increasing.

Many studies have confirmed that smoking can affect carbohydrate and lipid metabolism and increase the prevalence of T2DM [8, 9]. The number of intensive studies investigating various potential pathogenic factors and studies related to passive smoking as an environmental pathogenic factor of T2DM are increasing. A meta-analysis including seven prospective studies with a total of 162001 research subjects showed that passive smoking is a risk factor for T2DM [10]. To some extent, these results prove that both active and passive smoking have similar effects on the prevalence of T2DM. However, the effects of passive smoking on the prevalence of impaired fasting glucose (IFG) and impaired glucose tolerance (IGT) are unclear. Moreover, the effect of the duration of passive smoking on carbohydrate and lipid metabolism and the prevalence of IFG, IGT, and T2DM is unknown. Thus, we investigated these relationships in this study.

## RESULTS

Table 1 shows basic information such as age, body mass index (BMI), carbohydrate and lipid metabolism indexes of all participants and their comparisons between any two groups. There were no differences in age, BMI, and high-density lipoprotein (HDL) between the groups. There was no difference in any variable between Group A1 and Group B. However, low-density lipoprotein (LDL), total cholesterol (TC), triglyceride (TG), fasting plasma glucose (FPG), 2-hour postprandial glucose (2hPG), and glycated hemoglobin A1c (HbA1c) levels were significantly higher in Group A2 and Group A3 than in Group B (P<0.05). Moreover, LDL, TC, TG, FPG, 2hPG, and HbA1c levels were significantly higher in Group A2 and Group A3 than in Group A1 (P<0.05). There was no difference in any variables between Group A2 and Group A3. This suggested that passive smoking for >10 years aggravates carbohydrate and lipid metabolism.

**Table 1:** Basic information for all participants and comparison between groups.

	**All**	**Group B**	**Group A1**	**Group A2**	**Group A3**
N	3361	1326	1005	525	505
Age (years)	53.33±8.06	53.00±8.08	53.46±7.88	53.59±8.10	53.68±8.32
BMI kg/m^2^	23.61±3.36	23.54±3.20	23.51±3.40	23.70±3.24	23.91±3.78
HDL mmol/l	1.36±0.31	1.35±0.31	1.36±0.30	1.39±0.31	1.37±0.32
LDL mmol/l	2.81±0.79	2.74±0.80	2.75±0.74	2.99±0.80^*#^	2.94±0.79^*#^
TC mmol/l	4.87±1.00	4.74±1.01	4.77±0.94	5.15±0.98^*#^	5.09±1.00^*#^
TG mmol/l	1.45±1.04	1.40±0.94	1.38±1.08	1.61±1.18^*#^	1.57±1.02^*#^
FPG mmol/l	5.47±1.14	5.35±0.93	5.43±1.20	5.62±1.19^*#^	5.71±1.40^*#^
2hPG mmol/l	7.62±2.92	7.38±2.79	7.39±2.68	8.07±3.17^*#^	8.23±3.31^*#^
HbA1c %	5.84±0.77	5.76±0.60	5.77±0.79	6.01±0.93^*#^	6.01±0.85^*#^

Abbreviation: N: Number; BMI: body mass index; HDL-c: high-density lipoprotein cholesterol; LDL-c: low-density lipoprotein cholesterol; TC: total cholesterol; TG: triglyceride; FPG: fasting blood glucose; 2hPG: 2-hour postprandial glucose; HbA1c: glycated hemoglobin; All: All participants.

Data presented as mean ± SD.

*: Comparison between Group A1, Group A2, Group A3, and Group B (P<0.05).

#: Comparison between Group A2, Group A3, and Group A1 (P<0.05).

Table 2 shows the prevalence of IFG, IGT, and T2DM in each group and the comparisons between passive smoking groups and Group B. The prevalence of IGT was significantly higher in Group A (26.6%), Group A2 (28%), and Group A3 (37.8%) than in Group B (19.6%) (P<0.05). Moreover, the prevalence of IGT gradually increased in Group A1 (20.2%), Group A2 (28%), and Group A3 (37.8%). There were no differences in the prevalence of IFG and T2DM between the groups. Therefore, passive smoking increases the prevalence of IGT in a time-dependent manner, while no similar relationship was observed in IFG and T2DM.

**Table 2:** Prevalence of prediabetes and type 2 diabetes mellitus in each group.

**Diagnosis**	**Group B (N=1326)**	**Group A (N=2035)**	**P value**	**Group A1 (N=1005)**	**P_1_ value**	**Group A2 (N=525)**	**P_2_ value**	**Group A3 (N=505)**	**P_3_ value**
IFG	27 (2%)	53 (2.6%)	0.158	22 (2.2%)	0.799	17 (3.2%)	0.126	14 (2.8%)	0.341
IGT	260 (19.6%)	541 (26.6%)	<0.001	203 (20.2%)	0.723	147 (28%)	<0.001	191 (37.8%)	<0.001
T2DM	143 (10.8%)	229 (11.3%)	0.582	95 (9.5%)	0.293	70 (13.3%)	0.121	64 (12.7%)	0.254

Abbreviation: IFG: impaired fasting blood glucose; IGT: impaired glucose tolerance; T2DM: type 2 diabetes mellitus; N: number.

P: Comparison between Group A and group B.

P_1_: Comparison between Group A1 and group B.

P_2_: Comparison between Group A2 and group B.

P_3_: Comparison between Group A3 and group B.

Table 3 shows the results of multivariate logistic regression analyses. After adjusting for possible confounding factors, we found that when the duration of passive smoking is <10 years, it does not affect the prevalence of IFG, IGT, and T2DM. However, when the duration of passive smoking is >10 years, it is a risk factor for IFG (odds ratio [95% confidence interval]: Group A1: 1.07 [0.60–1.89], Group A2: 1.94 [1.04–3.61], and Group A3: 1.97 [1.02–3.82]), IGT (Group A1: 1.02 [0.83–1.26], Group A2: 1.74 [1.36–2.23], and Group A3: 2.78 [2.18–3.53]), and T2DM (Group A1: 0.86 (0.65–1.14), Group A2: 1.51 [1.09–2.08], and Group A3: 1.64 [1.17–2.30]). Moreover, an increase in the duration of passive smoking increased its effect on IFG, IGT, and T2DM, and the difference was significant when the duration was >10 years (P<0.05).

**Table 3:** Odds ratios and adjusted odds ratios for the prevalence of IFG, IGT, and T2DM in each group calculated using multivariate logistic regression analyses.

**Diagnosis**	**Groups**	**N/Total**	**OR (95% CI)**	**Adjusted OR (95% CI)**	
				**Model 1**	**Model 2**
IFG	Group B	27/1326	1	1	1
	Group A	53/2035	1.45(0.91–2.33)	1.45(0.91–2.33)	1.45(0.91–2.33)
	Group A1	22/1005	1.07(0.60–1.89)	1.06(0.60–1.89)	1.07(0.60–1.89)
	Group A2	17/525	1.94(1.04–3.61)*	1.94(1.04–3.61)*	1.94(1.04–3.61)*
	Group A3	15/505	1.97(1.02–3.81)*	1.97(1.02–3.81)*	1.97(1.02–3.82)*
					
IGT	Group B	260/1326	1	1	1
	Group A	541/2035	1.54(1.30–1.83)***	1.54(1.30–1.83)***	1.13(1.10–1.16)***
	Group A1	203/1005	1.02(0.83–1.26)	1.02(0.83–1.26)	1.02(0.83–1.26)
	Group A2	147/525	1.74(1.37–2.22)***	1.74(1.37–2.22)***	1.74(1.36–2.23)***
	Group A3	191/505	2.78(2.20–3.53)***	2.79(2.21–3.54)***	2.78(2.18–3.53)***
					
T2DM	Group B	143/1326	1	1	1
	Group A	229/2035	1.19(0.94–1.48)	1.19(0.95–1.49)	1.17(0.93–1.47)
	Group A1	95/1005	0.87(0.66–1.15)	0.87(0.66–1.15)	0.86(0.65–1.14)
	Group A2	70/525	1.51(1.10–2.07)*	1.51(1.10–2.07)*	1.51(1.09–2.08)*
	Group A3	64/505	1.70(1.22–2.36)*	1.70(1.23–2.36)**	1.64(1.17–2.30)**

Abbreviation: IFG: impaired fasting blood glucose; IGT: impaired glucose tolerance; T2DM: type 2 diabetes mellitus; OR: Odds Ratio; CI: Confidence Interval; N: number.

Model 1: Adjusted for the age;

Model 2: Adjusted for the age and BMI.

*: P<0.05;

**: P<0.01;

***: P<0.001.

## DISCUSSION

The development of health science has improved our understanding of factors that lead to disease. Researchers have studied the toxic effects of passive smoking as early as 1964 [11]. Similar to active smoking, passive smoking mostly affects the respiratory system. Therefore, researchers have focused mainly on the effects of passive smoking on respiratory diseases such as bronchitis, cancer, asthma, and early childhood pneumonia [12–14]. In 1978, Aronow WS et al. suggested that passive smoking can promote angina [15]. Since then, researchers have focused on the effect of passive smoking on other conditions and diseases such as platelet function, cognition, atherosclerosis, and rheumatoid arthritis [16–19].

In 1991, Feldman J et al. reported that passive smoking can affect lipid metabolism in adolescents [20]. Subsequently, several studies related to endocrine and metabolic diseases were reported [8, 21]. However, the results from studies on the effects of passive smoking on the prevalence of prediabetes and T2DM have been inconsistent. Some studies suggest that passive smoking can worsen carbohydrate and lipid metabolism disorders and promote the occurrence of T2DM [22–24], whereas other studies found that passive smoking had no effect on carbohydrate and lipid metabolism [8, 24]. However, so far, there has been no study on the relationship between the duration of passive smoking and the prevalence of prediabetes and T2DM. Therefore, further research is needed to explore the relationship among passive smoking, duration of passive smoking, and prevalence of prediabetes and T2DM.

Our results showed that passive smoking increases the prevalence of IGT in a time-dependent manner, while no similar relationship was observed in IFG and T2DM. When the duration of passive smoking is ≤10 years, there is no effect on carbohydrate and lipid metabolism and the prevalence of prediabetes and T2DM. However, when the duration of passive smoking exceeds 10 years, it may aggravate carbohydrate and lipid metabolism disorders, and is a risk factor for IFG, IGT, and T2DM, moreover, there was a time-dependent relationship between the duration of passive smoking and its effect on IFG, IGT, and T2DM. These results are consistent with those of previous studies [25, 26], although previous studies did not explore the effect of passive smoking duration [10, 26–28].

Nicotine and tar are the two main ingredients in tobacco that cause maximum damage to the human body, and these could be the harmful components of passive smoke. In addition, the health effects of passive smoke are related to ventilation, temperature, humidity, depth of breathing, and distance from the smoker. After a temperature drop and precipitation, some substances in the environment will agglutinate and damage the human body differently compared to active smoking [29, 30].

Regarding the mechanism by which passive smoking promotes carbohydrate and lipid metabolism disorders and increases the prevalence of IGT and T2DM, some studies have suggested that nicotine affects the function of islet cells and insulin [31–33]. Nicotine can activate AMP-activated protein kinase α2 in adipocytes to increase lipolysis of adipose tissue. Ultimately, nicotine promotes the degradation of the insulin receptor substrate-1 (IRS-1) and the loss of insulin-mediated lipolysis inhibition [34, 35]. Basic research suggested that IRS-1 serine phosphorylation was linked to insulin resistance [35]. Bergman et al. found that the increased phosphorylation of Ser636 in the serum of smokers and the activation mammalian target of rapamycin and mitogen-activated protein kinase signaling promote insulin resistance [36]. Moreover, smoking can cause endogenous stress responses leading to an increase in glycemic hormones (such as cortisol, catecholamines, and growth hormone) [37]. Through these mechanisms, smoking perturbs the carbohydrate and lipid metabolism and promotes the development of an abnormal glucose metabolism.

This is the first study to investigate the effects of the duration of passive smoking on the prevalence of prediabetes and T2DM. In addition, we reviewed the existing literature. However, this study had many limitations. First, because this was a cross-sectional study, it can explain the correlation between passive smoking and the prevalence of prediabetes and T2DM but not causality. Second, the study participants were all women; thus, the results do not represent the overall population. Third, the definition of passive smoking was based on the results of the questionnaire without quantitative indicators. Fourth, it is difficult to accurately evaluate ventilation, temperature, humidity, depth of breathing, and distance between passive smokers and smokers. These issues may partially affect the results.

## CONCLUSIONS

Passive smoking increases the prevalence of IGT in a time-dependent manner, however, when the duration of passive smoking exceeds 10 years, it may aggravate carbohydrate and lipid metabolism disorders, and is a risk factor for IFG, IGT, and T2DM, moreover, there was a time-dependent relationship between the duration of passive smoking and its effect on IFG, IGT, and T2DM.

## MATERIALS AND METHODS

### Participants

This cross-sectional study included 3361 non-smoking women (age 25–75 years, mean age: 53.33 ± 8.06 years). They were selected using a stratified random sampling method between May 2017 and February 2019 from 10 urban and rural areas in the Jiangxi Province.

### Definitions

Data regarding passive smoking (such as duration and frequency) were collected using self-reported questionnaires. Participants were placed in the passive smoking group (Group A) if they were exposed to cigarette smoke for ≥15 minutes/day, ≥3 days/week, and ≥1 year. Otherwise, participants were included in the control group (Group B). Furthermore, according to the duration of passive smoking, Group A was divided into Group A1 (≤10 years), Group A2 (10–20 years), and Group A3 (>20 years). The definitions of prediabetes and T2DM are based on the diagnostic and classification criteria reported by the 1999 World Health Organization Expert Committee on Diabetes [38]. The definition of T2DM was diabetic symptoms and a random blood glucose ≥11.1 mmol/l or fasting (no calorie intake for ≥8 hours) blood glucose ≥7.0 mmol/l and oral glucose tolerance test (OGTT) at 2-hour postprandial glucose (2hPG) ≥11.1 mmol/l. The definition of IGT was fasting plasma glucose (FPG) <7 mmol/l and OGTT at 2hPG ≥7.8 mmol/l but <11.1 mmol/l. The definition of IFG was FPG ≥6.1 mmol/l but <7.0 mmol/l and OGTT at 2hPG <7.8 mmol/l. Normal blood glucose was defined as FPG <6.1 mmol/l and OGTT at 2hPG <7.8 mmol/l. T2DM patients in this study were considered to belong to one of the following scenarios: 1) diagnosed as T2DM or requiring hypoglycemic drugs or insulin or 2) undiagnosed but with FPG or 2hPG levels that surpassed the ranges of the T2DM diagnostic criteria.

### Anthropometric and carbohydrate and lipid metabolism indexes

The researchers involved in this study were professionally trained. The measurements of height and weight were recorded when the participants were wearing light clothing without hats or shoes. The measurement tools were calibrated, and the average of three measurements was considered as the final result. BMI was calculated using the height and weight (kg/m^2^). The carbohydrate and lipid metabolism indexes included HDL, LDL, TC, TG, FPG, 2hPG, and HbA1c. Participants with T2DM and those without T2DM were administered 100 g of steamed bread and 75 g of anhydrous glucose powder, respectively, before the 2-h postprandial blood test for measuring 2hPG.

All data, including age, BMI, HDL, LDL, TC, TG, FPG, 2hPG, and HbA1c, were collected and sorted using Excel (Microsoft Office 2019) and imported into SPSS version 23.0 (International Business Machines Corporation). Data (presented as mean ± SD) between two groups were compared using the Student-Newman-Keuls method of analysis of variance. Cochran’s and Mantel-Haenszel statistics were used to compare the prevalence of IFG, IGT, and T2DM between passive smoking groups and control group. Multivariate logistic regression analyses were used to control confounding factors.
